# Supplementary material for: Content-rich biological network constructed by mining PubMed abstracts
Source: BMC Bioinformatics. 2004 Oct 8;5:147. doi: 10.1186/1471-2105-5-147 (PMC528731; doi:10.1186/1471-2105-5-147)
Supplement: Additional File 2 — The original results of the above study (non-essential files are deleted to keep the file size under the limit set by BMC bioinformatics). [file 1471-2105-5-147-S2.bz2 › chilibotAdditionalFile2/dip10/277ID1827203E1097/html/ATF3_JUN.html]

 


 **ATF3** and **JUN** 
  
Found 44 abstracts in PubMed, retrieved 10.  
 

 What does Google say? 
 PDF only 
| .edu only 

---

**Interactive relationship** (e.g. stimulation, inhibition, etc)

**Neutral relationship**- Activating transcription factor 3  [ **ATF3** ]   **ATF3**  is a basic leucine zipper protein which is highly homologous to c  **Jun**  dimerization protein 2 JDP2, especially within the threonine proline phosphoacceptor site, Thr 148.  Ref: 12225289 Biochem J, 2002

**Non-interactive relationship** (e.g. studied together, co-existance, homology, etc.)

- Electrophoretic mobility shift assays identified Nrf2, MafG,  **ATF3** , and  **Jun**  and Fos family members as StRE binding proteins.  Ref: 12453873 Am J Physiol Renal Physiol, 2003
- Using in situ hybridization and immunohistochemistry, we compared the expression profile of ATF 3  [ **ATF3** ]  with those of ATF 2 and c  **Jun**  after middle cerebral artery MCA occlusion.  Ref: 12877985 Brain Res Mol Brain Res, 2003
- We conclude that the combination of  **ATF3**  and c  **Jun**  induces the anti apoptotic factor Hsp27, which directly or indirectly activates Akt, and thereby possibly inhibits apoptosis and induces nerve elongation.  Ref: 12832543 J Neurosci, 2003
- Expression of the activating transcription factor 3  [ **ATF3** ]  prevents c  **Jun**  N terminal kinase induced neuronal death by promoting heat shock protein 27 expression and Akt activation.  Ref: 12832543 J Neurosci, 2003
- Among the 41 statistically significant named overexpressed genes at the 1 hour time point were early growth response 1 EGR1,  **jun**  B proto oncogene  **jun**   [ **JUN** ]  B,  **jun**  D proto oncogene  **jun**   [ **JUN** ]  D, and activating transcription factor 3  [ **ATF3** ]   **ATF3** .  Ref: 12414521 Am J Pathol, 2002
- This cluster included genes encoding stress related proteins e.g.  **Jun** ,  **ATF3** , TAB1, and TANK, suggesting that their up regulation may be in response to secondary necrosis.  Ref: 12488458 J Biol Chem, 2003
- The expression profiles of ATF 3  [ **ATF3** ]  and c  **Jun**  were virtually similar, but c  **Jun**  expression was also observed in other regions of the brain in control rats.  Ref: 12877985 Brain Res Mol Brain Res, 2003
- Immunoprecipitation analysis and promoter assay for Hsp27 expression suggest that both  **ATF3**  and c  **Jun**  are necessary for transcriptional activation of Hsp27.  Ref: 12832543 J Neurosci, 2003
- cooperative works of ATF 3  [ **ATF3** ]  and c  **Jun**  may be crucial triggers of various transcriptional responses to the ischemic insult.  Ref: 12877985 Brain Res Mol Brain Res, 2003
- IR was found to enhance the expression of c  **Jun**  and, in particular,  **ATF3** , but, in contrast to various other stress stimuli, did not induce the expression of c Fos.  Ref: 12833146 Oncogene, 2003
- Overexpression of  **ATF3**  by adenovirus inhibits the mitogen activated kinase kinase kinase 1 MEKK1 c  **Jun**  N Terminal Kinase JNK induced apoptosis and induces neurite elongation via Akt activation in PC12 cells and superior nerve ganglion neurons.  Ref: 12832543 J Neurosci, 2003
- Double immunohistochemical labeling revealed concurrent expression of ATF 3  [ **ATF3** ]  and phospho c  **Jun**  in neurons.  Ref: 12877985 Brain Res Mol Brain Res, 2003
- Our results suggest that  **ATF3**  and c  **Jun**  induced Hsp27 expression is a novel survival response in neurons under death stress such as nerve injury.  Ref: 12832543 J Neurosci, 2003
- DOX rapidly activated JNK and c  **Jun**  and induced  **ATF3**  at both mRNA and protein level.  Ref: 12392999 J Mol Cell Cardiol, 2002
